# Ambulante Phonochirurgie

**DOI:** 10.1007/s00106-021-01081-6

**Published:** 2021-06-30

**Authors:** Jörg E. Bohlender

**Affiliations:** Abteilung Phoniatrie und Klinische Logopädie, Klinik für Ohren‑, Nasen‑, Hals- und Gesichtschirurgie, UniversitätsSpital Zürich, Universität Zürich, Frauenklinikstr. 24, 8091 Zürich, Schweiz

**Keywords:** Ambulante chirurgische Eingriffe, Endoskopie, Lasertherapie, Larynx, Patientensicherheit, Ambulatory surgical procedures, Endoscopy, Laser therapy, Larynx, Patient safety

## Abstract

Wesentliche und neue Impulse, die das Behandlungskonzept für ambulante phonochirurgische Eingriffe am unsedierten Patienten betreffen, stammen aktuell aus dem angloamerikanischen Sprachraum. Die dort etablierten „office-based procedures“ werden als eine Alternative zu vielen konventionellen operativen Larynxeingriffen in Vollnarkose propagiert. Maßgeblich für diese Entwicklung ist der Einsatz neuer endoskopischer Techniken in Kombination mit photoangiolytischen Laserverfahren (KTP-Laser und blauer Laser), die eine sichere und effiziente ambulante Phonochirurgie ermöglichen. Die Akzeptanz seitens der Patienten ist hoch, da ambulante Eingriffe als risikoärmer gelten. Ungeachtet der verbreiteten Euphorie fehlen weiterführende Studien, welche die medizinischen Entscheidungskriterien und das Sicherheitsmanagement bei dieser neuen ambulanten Ausrichtung der Kehlkopfchirurgie bewerten.

## Abstract (en)

Significant and new impulses regarding the treatment concept for outpatient phonosurgery on unsedated patients are currently coming from various Anglo-American authors. These “office-based procedures” have been propagated as an alternative to many conventional surgical interventions under anesthesia. The main reason for this remarkable development is the use of new endoscopic techniques in combination with photoangiolytic laser (KTP and blue laser), which allow safe and efficient phonosurgical procedures. Patient acceptance is high since outpatient procedures are considered to have a lower surgical risk. Despite the widespread euphoria, there is a lack of studies evaluating the medical decision criteria and safety management of office-based laryngeal surgery.

Die ambulante Kehlkopfchirurgie im Behandlungsstuhl ohne Vollnarkose wird routinemäßig seit mehr als 20 Jahren in vielen laryngologischen Zentren der USA als chirurgische Therapiealternative angeboten. Diese als „in-office laryngeal procedures“ (IOLP) bezeichnete Interventionsform nutzt die Weiterentwicklung der flexiblen Endoskopie mit ihrer hochauflösenden Bildqualität in Kombination mit neuartigen technischen Möglichkeiten an phonochirurgischen Eingriffen. So lassen sich über einen integrierten Arbeitskanal Lasereingriffe, Abtragungen und Injektionen bei unterschiedlichen laryngealen Pathologien erfolgreich durchführen.

Als medizinhistorischer Meilenstein in der Laryngologie gilt die erstmals 1861 von Victor von Bruns (1812–1883) durchgeführte transorale Exstirpation eines Kehlkopfpolypen. So schreibt der in Tübingen an der Universität lehrende Chirurg von Bruns im Kapitel *Allgemeine Bemerkungen über laryngoskopische Operationen und Instrumente *in seiner 1862 erschienenen Publikation „Die erste Ausrottung eines Polypen in der Kehlkopfshöhle durch Zerschneiden ohne blutige Eröffnung der Luftwege nebst einer kurzen Anleitung zur Laryngoskopie“ [1], dass „an den operi(e)renden Arzt, wie an den zu operi(e)renden Kranken eigent(h)ümliche Anforderungen“ gestellt werden und dass „Operationen an solcher Stelle ohne allen Widerspruch zu den schwierigsten in dem ganzen grossen Gebiete der Chirurgie“ zählen (Abb. 1). Mit der Entwicklung und Etablierung der Intubationsnarkose und Einzug des Operationsmikroskops inklusive Stützlaryngoskopie sowie der Implementierung verschiedener Operationstechniken wurden ambulante transorale Kehlkopfeingriffe im Behandlungsstuhl nur noch von einigen wenigen Laryngologen regelmäßig praktiziert. Dank der technologischen Weiterentwicklung der flexiblen Endoskopie in Kombination mit neuartigen Laserverfahren steht eine deutlich größere Bandbreite an ambulanten Therapieoptionen im Bereich des Larynx zur Verfügung. Für den chirurgisch tätigen Laryngologen bedeutet dies, dass sich das ambulante Behandlungskonzept nicht mehr allein auf Patienten mit ungenügender Einstellbarkeit im Rahmen einer Stützlaryngoskopie (z. B. Trismus, Retrognathie, prominente Schneidezähne) oder Patienten mit Komorbiditäten, die keinen Eingriff in Vollnarkose erlauben, beschränkt. Die zunehmende Präferenz und Akzeptanz der Patienten für „office-based“ Eingriffe ohne Vollnarkose ergänzen die allgemein bekannten Vorteile: Kostenersparnis, ein kürzeres Zeitintervall von der Diagnose bis zur Therapie, keine Hospitalisierung und Verminderung potenzieller nosokomialer Infektionen.

**Figure 1:**
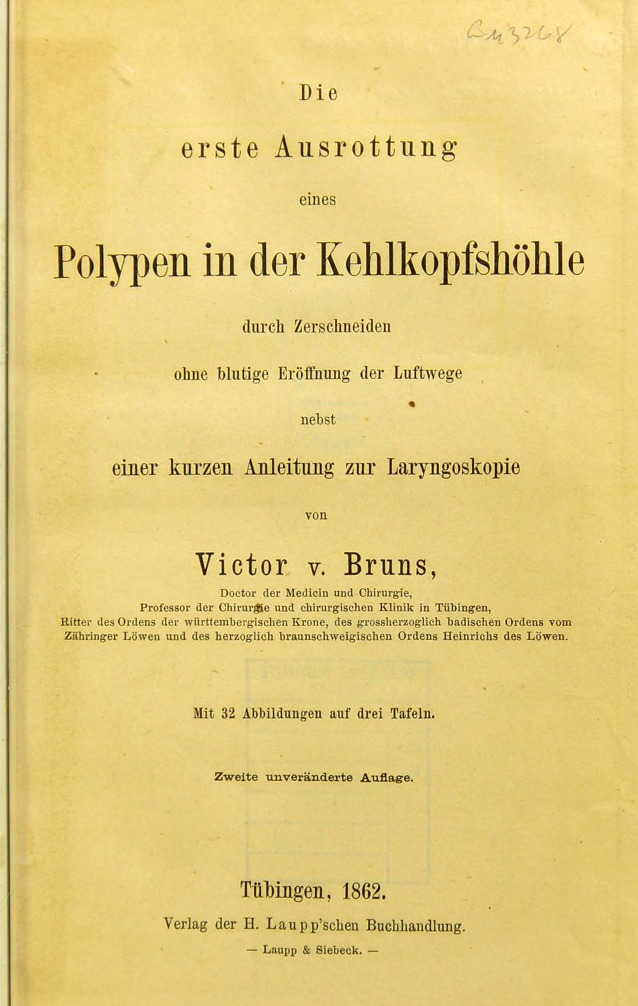

Ungeachtet der in vielfachen Publikationen verbreiteten Euphorie fehlen nach wie vor umfassende klinische Studien und Richtlinien, welche die Abläufe und das Sicherheitsmanagement bei diesen Eingriffen kritisch begleiten. Auch wenn die Komplikationen bei laryngealen Eingriffen am wachen Patienten nach wie vor eher als gering eingestuft werden, bestehen potenzielle Risiken. Bekannt sind u. a. vasovagale Reaktionen, Laryngospasmen, Einblutungen, allergische Reaktionen und die Gefahr einer Überdosierung von Lidocain [19]. Aus eigener langjähriger operativer Erfahrung (> 100 „office-based“ Eingriffe pro Jahr) und nach Literaturdurchsicht lassen sich keine gravierenden Komplikationen nennen.

Die Entscheidung für einen „office-based“ Eingriff ohne Narkose ist stets eine Einzelfallentscheidung

Der behandelnde Arzt muss die Patientensicherheit und damit das Management unerwarteter intra- und postoperativer Komplikationen bei dieser Art von Chirurgie am wachen Patienten garantieren. In unserem klinischen Alltag wird im Bedarfsfall bei älteren und kardiopulmonal vorbelasteten Patienten ein intravenöser Zugang inklusive Monitoring empfohlen. Weiterhin werden Patienten, bei denen ein kritischer Verlauf möglich ist, postoperativ beobachtet. Die Entscheidung für einen „office-based“ Eingriff ohne Narkose ist daher immer eine Einzelfallentscheidung.

## Voraussetzungen

Der behandelnde Arzt kann schon im Rahmen der Diagnostik und im Vorfeld der Planung eventuelle anatomische Schwierigkeiten und eine erhöhte Reflexbereitschaft einschätzen. Eine sorgfältige Indikationsstellung für den phonochirurgischen Eingriff sollte jenseits der verfügbaren Therapieoptionen die individuellen Bedürfnisse, die Behandlungsmotivation und den Gesundheitszustand des Patienten berücksichtigen (Infobox 1).

### Infobox 1 Checkliste „ambulante Phonochirurgie“

- Sorgfältige Auswahl des zu behandelnden Falls erfolgt?
  - Patient für den geplanten Eingriff geeignet?
  - Läsion in einer angemessenen Zeit komplett resektabel?
- Operationsaufklärung erfolgt?
- Prä-, intra- oder postoperative Überwachung notwendig?
- Risiken/Notfälle managebar?
- Intraoperativ ggf. alternative Zugangswege erforderlich?
- COVID19-Abstrich negativ?

Phonochirurgische Eingriffe im Wachzustand sollten nicht als Gelegenheitschirurgie verstanden werden

**Figure 2:**
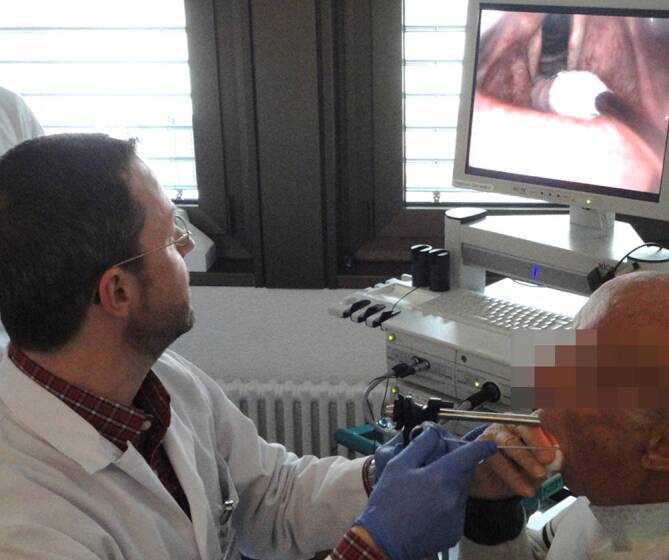

Ein ambulantes Vorgehen in Oberflächen‑/Lokalanästhesie muss den qualitativen Ansprüchen einer Operation im Operationssaal entsprechen. Dies setzt ein spezifisch manuell chirurgisches Geschick, kontinuierliches Training und Expertise des Operateurs voraus. Im Idealfall sollte der Behandler über ein breites Repertoire an ambulanten phonochirurgischen Techniken verfügen. Es wäre aus Sicht des Autors zu begrüßen, wenn im Zuge des technischen Fortschritts der flexiblen Endoskopie auch die verkannten Vorteile der transoralen indirekten Technik eine Neubewertung erfahren würden. Gerade Injektionen und Biopsien lassen sich bei großer Akzeptanz der Patienten in der Regel problemlos transoral und sicher im Larynx durchführen. Dies setzt einen routinierten Umgang mit starren Optiken (70° und 90°) voraus (Abb. 2).

Eine prä- und posttherapeutische Stimmfunktionsdiagnostik, vorzugsweise nach dem ELS(European Laryngological Society)-Protokoll, erfasst den funktionalen Anteil einer benignen Stimmlippenpathologie und trägt zur Qualitätssicherung und Beurteilung des Outcomes von phonochirurgischen Maßnahmen bei [3]. Eine differenzierte Befunderhebung und Dokumentation der Stimmstörungen wird jedoch in der gängigen internationalen Literatur bedauerlicherweise häufig nur rudimentär reflektiert, obgleich die Chirurgie als „phonochirurgisch“ gelabelt wird.

## Vorgehen

Laryngeale Eingriffe im Stuhl sollten zunächst unabhängig von den geplanten Interventionen und der Dauer nie alleine durchgeführt werden. Aus eigener Erfahrung hat sich eine Tandemsituation (Operateur und Assistenz) im klinischen Alltag bewährt. Vor jedem Eingriff muss eine kritische Prüfung der Technik, der Endoskope, des Saugers und vor allem des Instrumentariums (Laser oder mikrochirurgische Instrumente) erfolgen. Bei Lasereingriffen im Behandlungsstuhl sollten die empfohlenen allgemeinen Sicherheitsvorkehrungen getroffen werden.

Die gegenwärtige SARS-CoV-2(„severe acute respiratory syndrome coronavirus 2“)-Pandemie stellt auch für den operativ tätigen Laryngologen eine immense Herausforderung dar. Gerade bei den transoralen und transnasalen aerosolgenerierenden Eingriffen ist ein Höchstmaß an Selbst‑, Mitarbeiter- und Patientenschutz erforderlich, sie hat höchste Priorität bei der Planung von Operationen. Die Teamarbeit bewährt sich bei der gegenseitigen Kontrolle der Schutzkleidung und Ausrüstung sowie beim Einhalten der Hygienemaßnahmen. Vor „office-based“ Eingriffen ist ein negativer COVID(„coronavirus disease“)19-Test des Patienten unabdingbar.

### Aufklärung und Setting

Der Patient darf erwarten, dass er grundsätzlich in einer ruhigen und professionellen Atmosphäre behandelt wird. Die präoperative Aufklärung sollte idealerweise einige Tage zuvor durchgeführt worden sein. Der Patient kennt damit bereits das Operationsteam, was sich im weiteren Umgang als Vorteil erweist. Zu Beginn werden noch etwaige offene Fragen geklärt. Wird die Operation sitzend im Behandlungsstuhl durchgeführt, sollte auf eine angenehme und entspannte Sitz- und Kopfposition geachtet werden. Die einzelnen operativen Schritte werden von Beginn an verbal begleitet. Gerade die Oberflächenanästhesie im Mundrachenraum und im Larynxbereich wird von den Patienten häufig als die eigentlich kritische und teilweise bedrohliche Phase erlebt. Die subjektive Unfähigkeit zu schlucken, das fehlende Gespür für den Atemstrom und ein kurzfristig auftretender Hustenreiz dürfen vom Behandlungsteam nicht bagatellisiert werden. Der Operateur bestätigt diese vom Patienten geäußerten Sensationen als eine adäquate Reaktion. Die Aufforderung, die reflektorisch verstopfte Nase zu schnäuzen, die Nasenatmung kurz anzuwenden und anschließend leer zu schlucken, sind einfache und hilfreiche Handlungsempfehlungen.

Ein innovatives und vielversprechendes Laserverfahren stellt die selektive Photoangiolyse (SPA) dar

Unabhängig vom gewählten Zugang muss bei der Planung des Eingriffs die kurze Wirkdauer des topisch applizierten Lokalanästhetikums (ca. 15 min) bei der Operationsplanung berücksichtigt werden.

Hierbei bieten sich verschiedene Verfahren in Abhängigkeit zur Pathologie an.

### Instrumente

Weitgehend unverändert sind die seit Jahrzehnten in ihrer Form und Funktion zum Einsatz kommenden mikrochirurgischen Instrumente für transorale indirekte Eingriffe.

**Figure 3:**
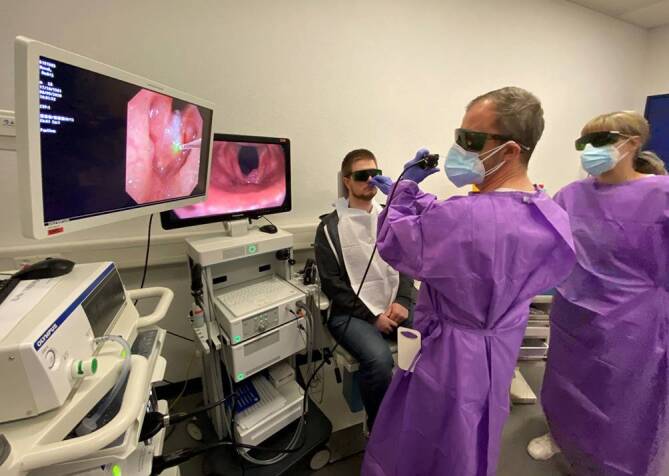

Mit der Weiterentwicklung der flexiblen Endoskopietechnik können mittlerweile unterschiedliche miniaturisierte mikrochirurgische Instrumente, aber auch Laserfasern in den Arbeitskanal integriert werden. Ein innovatives und vielversprechendes Laserverfahren stellt die selektive Photoangiolyse (SPA) dar. Hierbei interagiert selektives photoangiolytisches Laserlicht mit dem roten Blutfarbstoff (Hämoglobin) und führt zu einer selektiven Verödung von kleinsten Mikrogefäßen in der Stimmlippe. Mit dieser kontaktfreien Lasertechnik können beispielsweise laryngeale Papillome unter Schonung der empfindlichen Stimmlippenarchitektur gezielt und effizient endoskopisch behandelt werden (Abb. 3). Selektives photoangiolytisches Laserlicht wie Kalium-Titanyl-Phosphat (KTP; 532 nm) oder der „blaue Laser“ (445 nm) erweitern damit die bisherigen therapeutischen Optionen bei Eingriffen im Behandlungsstuhl [11]. Bei allen Vorteilen, welche die photoangiolytische Lasertechnik im „office-based“ Setting mit sich bringt, müssen potenzielle Risiken schwankender Laserenergieabgaben bei mobilen Stimmlippen und gleichzeitig veränderbarer Position der Laserspitze weiterhin geklärt werden. Die Anwendungsmöglichkeiten des nun auch in der flexiblen Endoskopie kommerziell zur Verfügung stehenden CO_2_(Kohlenstoffdioxid)-Lasers bleiben noch zu evaluieren [18].

### Gutartige Stimmlippentumoren

Eine Vielzahl an gutartigen Läsionen lässt sich sicher und effizient ohne Narkose im Wachzustand behandeln (Tab. 1).

**Table 1:** 

Pathologie	Phonochirurgisches Verfahren	Anmerkungen
Larynxpapillomatose	SPA	Ideale Pathologie für Lasertherapie
		Biopsie und HPV-Subtypisierung notwendig
		Ideal auch für mehrfache Eingriffe bei Rezidivneigung
		Größere Papillomvolumina bei begrenzter Operationsdauer nicht in toto abtragbar
		Kombination mit Cidofovir oder Bevazicumab-Injektionen
Stimmlippenpolyp	SPA in Kombination oder allein mit mikrochirurgischer Abtragung	Bei alleiniger Laserabtragung keine Histologie möglich
	Transorale Abtragung in toto	Weniger gut geeignet bei größeren Stimmlippenpolypen
		Hämorrhagische Stimmlippenpolypen für Laserbehandlung ideal
		Kleine Stimmlippenpolypen auch transoral geeignet
Exsudative Läsionen des Reinke-Raums (u. a. Reinke-Ödem, Pseudozyste, „weiche“ Stimmlippenknötchen)	SPA	Tendenziell eher für glasig-ödematöse Läsionen geeignet
	Transoral	Organisierte derbe Läsionen für Laser weniger geeignet
		Zurückhaltend bei größeren Läsionen, die den Atemweg verlegen (Cave: Zunahme der Schwellung nach Laserbehandlung)
		Funktionelles Resultat erst nach einigen Wochen beurteilbar
		Bei kleineren umschriebenen Läsionen transoral
		Intraläsionale Kortikoid-Injektionen zur Volumenreduktion
Vaskuläre Malformationen	Transnasal endoskopisch mit KTP-Laser oder „blauer Laser“	Ideal für Laserbehandlung
		Einblutung muss vermieden werden
Stimmlippenzysten	SPA	Keine vollständige Exstirpation!
		Rezidivgefahr
Kontaktgranulom	SPA	Operatives Vorgehen nur bei Zunahme und Rezidiven empfohlen
		Zusätzlich: Botulinumtoxininjektionen oder Kortisoninjektionen in kleineren Studien beschrieben
		Injektionslaryngoplastik bei bestehender Glottisschlussinsuffizienz als Therapieoption prüfen
Narbige Stimmlippenveränderungen	SPA in Kombination mit Steroidinjektion	Vorteil der Laserbehandlung noch nicht abschließend geklärt
	Transorale Steroidinjektion	Bei lokal umschriebenen Veränderungen versuchsweise auch subläsionale Narbensprengungen mit isotonischer NaCl-Lsg.,um die Option einer späteren Injektionslaryngoplastik zu überprüfen
	Transnasale und transorale subläsionale Narbensprengung	
Leukoplakien, Erythroplakien Hyperkeratosen	Transorale oder transnasale subepitheliale Hydrodissektion und Abtragung	Biopsie sinnvoll
	SPA	Kleinere Läsionen transoral Exzision in toto möglich (Biopsie = Exzision)
		Subläsionale Laseranwendung potenziell effektiv
		Mehrere Eingriffe notwendig
		Bei auffällig chronisch laryngitisch veränderten Stimmlippenbelägen: transoraler „Abstrich“ für mikrobiologische Diagnostik
Spasmodische Dysphonie	*Neben perkutanen Zugangswegen v.* *a.*	Bei spasmodischer Dysphonie vom Adduktortyp geeignet
	Transnasal endoskopisch	Gute Visualisierung (auch Supraglottis)
	Transoral	Kein EMG zur korrekten Platzierung erforderlich
Glottisschlussinsuffizienz	*Neben perkutanen Zugangswegen v.* *a.*	Kein EMG zur korrekten Platzierung erforderlich
	(Transnasal endoskopisch)	Ideal für transorales Vorgehen unabhängig der Ätiologie (taktil-kinästhetische Kontrolle)
	Transoral	Transnasal endoskopische Injektionslaryngoplastik nur mit weniger viskösem Injektionsmaterial möglich
		Überschuss an Injektionsmaterial in der langen Nadel

*EMG *Elektromyographie, *HPV *humane Papillomaviren*, KTP *Kalium-Titanyl-Phosphat*, NaCl *Natriumchlorid*, SPA* selektive Photoangiolyse (mittels transnasaler endoskopischer Verfahren, z. B. mit dem KTP-Laser und dem „blauen Laser“)

Im Folgenden werden einzelne Befunde und Behandlungsstrategien diskutiert.

### Papillome

**Figure 4:**
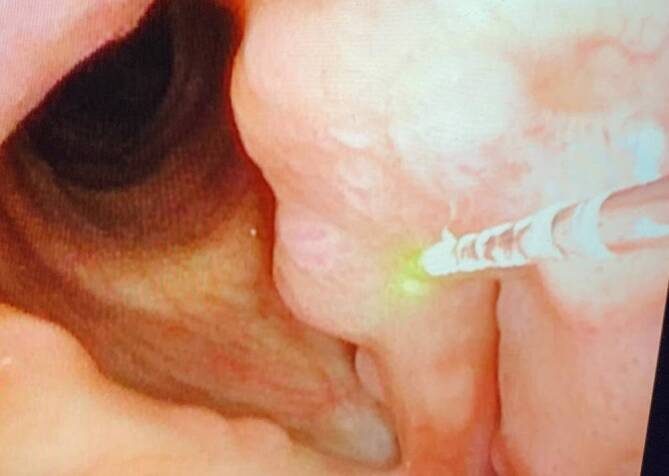

Der vaskuläre Charakter von laryngealen Papillomen stellt für eine photoangiolytische Laserbehandlung mit dem KTP oder blauen Laser eine ideale Pathologie dar (Abb. 4). Die vergleichbaren postoperativen funktionellen Ergebnisse bei einem ambulanten Vorgehen bestätigen sich in einem aktuellen Review und einer systematischen Metaanalyse [5]. In Kombination mit dem Laser lassen sich auch intraläsionale Cidofovir- oder Bevacizumabinjektionen durchführen.

### Stimmlippenpolyp

Die ambulante photoangiolytische Laserbehandlung von Stimmlippenpolypen wird mehr und mehr als eine vielversprechende Alternative zu einem Eingriff in Vollnarkose propagiert. Wie auch bei allen anderen „office-based“ Eingriffen in Lokalanästhesie ist die Operationszeit begrenzt. Bei bis zu 10 % der Patienten, beispielsweise mit größeren Polypen oder intraoperativen Beschwerden, kann der Eingriff nicht wie geplant beendet werden [13]. Einige Autoren empfehlen daher, die Laserkoagulation mit einer anschließenden endoskopischen Polypektomie des koagulierten Polypen zu verbinden. Diese Kombination soll weitere Behandlungssitzungen bei alleiniger Anwendung des photoangiolytischen Lasers vermeiden [17].

### Kontaktgranulom

Auch wenn unter Schonung des Perichondriums eine sorgfältige ambulante mikrochirurgische Abtragung in Kombination mit einer KTP-Lasertechnik erfolgt [8], muss mit einem Rezidiv des Kontaktgranuloms gerechnet werden. Nur bei einem suspekten Befund oder bei Verlegung des Glottisspalts empfiehlt sich die Chirurgie. Weiterhin ergänzen intraläsionale Injektionen von entzündungshemmenden Kortikosteroiden oder Botulinumtoxininjektionen zur Schwächung des schädigenden und verursachenden Hammer-Amboss-Prinzips das Therapieregime. Ungeachtet dieser diskussionswürdigen therapeutischen Maßnahmen sollte eine potenzielle Glottisschlussinsuffizienz als zugrundeliegende Ursache ausgeschlossen werden [4].

### Exsudative Läsionen

**Figure 5:**
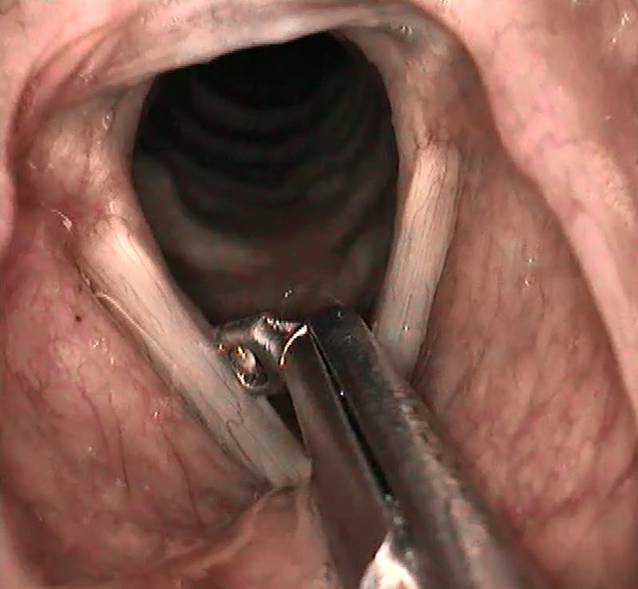

Die makroskopisch teilweise schwer voneinander zu unterscheidenden bzw. zu klassifizierenden benignen Stimmlippenveränderungen wie Reinke-Ödeme, Stimmlippenpolypen und seröse Pseudozysten zeigen eine histopathologische Gemeinsamkeit, die sich durch charakteristische exsudativen Läsionen im Reinke-Raum auszeichnet [10]. Neben einer ambulanten Abtragung mit kalten mikrochirurgischen Instrumenten (v. a. transoral indirekt; Abb. 5) werden transnasale Laserverfahren eingesetzt. Alternativ zu diesen genannten Verfahren werden bei „office-based“ Eingriffen Kortikosteroidinjektionen bei der Behandlung von sogenannten exsudativen Läsionen des Reinke-Raums empfohlen [6]. Teilweise gehen diese volumenreduzierenden Injektionen einem späteren phonochirurgischen Eingriff voraus. In den unterschiedlichen Studien werden sowohl Triamcinolon als auch Dexamethason monotherapeutisch oder in Kombination appliziert. Die Dosierung und die Behandlungsfrequenz unterscheiden sich hierbei deutlich von Studie zu Studie [6].

### Stimmlippenzsyten

Idealerweise werden bei der Dissektion von Zysten Mikroflap-Techniken angewandt. Mit Hilfe der transnasal endoskopischen KTP-Laser-Technik wird eine Drainage und Marsupialisation von Retentionszysten empfohlen. Kritisch wird vermerkt, dass die angewandte Lasertechnik weniger präzise in der Anwendung sei und dass ein potenzielles Risiko zusätzlicher Narbenbildung bestehe [9]. Hier stellt sich die Frage, inwieweit dieses laserassistierte Vorgehen grundsätzlich mit dem phonochirurgischen Konzept einer funktionell orientierenden und gewebeschichtrespektierenden Chirurgie in Einklang zu bringen ist.

### Vaskuläre Malformationen

Die photoangiolytische Behandlung stellt bei mikrovaskulären Malformationen ebenfalls eine ideale Therapieoption dar. Der Operateur sollte dennoch bei der Therapieplanung sorgfältig in Betracht ziehen, dass bei fehlender Dokumentation einer Hämorrhagie in der Krankengeschichte, eine Laserbehandlung möglicherweise einen unnötigen Eingriff darstellt [16]. Die Ablation der vaskulären Malformationen erfolgt direkt oder indirekt an das umgebende Gebiet des Gefäßes. Die Laserbehandlung von kleinsten Stimmlippengefäßen beim wachen Patienten stellt für den Operateur eine Herausforderung dar, da eine Einblutung in das umliegende Stimmlippengewebe dringlichst vermieden werden sollte.

#### Leukoplakien, Erythroplakien und Hyperkeratosen

In laryngologischen Zentren werden zunehmend leukoplakische Alterationen, Erythroplakien und hyperkeratotische Veränderungen im Bereich der Stimmlippen trotz histopathologisch unterschiedlicher Dysplasiegrade photoangiolytisch erfolgreich therapiert [14]. Jenseits der Diskussionen um Zeit- und Kosteneffizienz besteht Diskussionsbedarf, inwieweit das ambulante Laserverfahren ohne vorangegangene Biopsie mit den onkologischen Kriterien einer zuverlässigen und sicheren Therapie vereinbar ist.

### Narben

**Figure 6:**
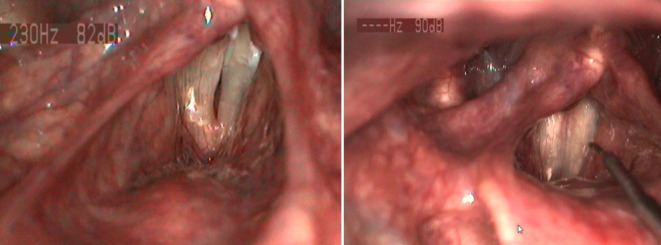

Ambulant kann mit Hilfe von intraläsionalen Steroidinjektionen eine Verbesserung der Stimmlippenbeweglichkeit und eine Verbesserung des Glottisschlusses verfolgt werden [7]. Bei kleineren überschaubaren Defekten kann eine ambulante subepitheliale Narbensprengung versucht werden (Abb. 6). So kann nach einer erfolgreichen Expansion des narbigen Defekts eine Folgeinjektion beispielsweise mit Hyaluronsäure, falls notwendig, geplant werden. Mittlerweile werden auch angiolytische Laseranwendungen bei narbigen Veränderungen diskutiert. Experimenteller Natur ist die gezielte ambulante Injektion von basischen Fibroblastenwachstumsfaktoren (bFGF) in die Stimmlippennarben [15].

### Spasmodische Dysphonien

„Office-based“ Injektionen von Botulinumtoxin stellen das ideale Setting bei der Behandlung einer spasmodischen Dysphonie dar, können aber auch bei essenziellem Stimmtremor durchgeführt werden [2, 12]. Die intramuskulären Injektionen lassen sich bei der spasmodischen Dysphonie vom Adduktortyp perkutan translaryngeal (transthyrohyoidal, transcricothyroidal, transthyroidal), transoral oder transnasal flexibel endoskopisch durchführen. Die perkutane translaryngeale Technik wird nach einer Lokalanästhesie im Bereich der Injektionsstelle in der Regel EMG(Elektromyographie)-gesteuert durchgeführt. Eine fehlende direkte visuelle Kontrolle und auch mangelnde präzise Injektion in das muskuläre Zielgebiet dürfen als Limitationen dieses Verfahrens gewertet werden. Der Vorteil einer direkten visuellen Kontrolle (flexibel endoskopisch oder indirekt transoral) erlaubt eine bilaterale Injektion in den M. thyroarytenoideus sowie in die Muskulatur der Taschenfalten. Der Zugangsweg zum M. cricoarytenoideus posterior erfolgt bei der spasmodischen Dysphonie vom Abduktortyp perkutan cricothyroidal-transglottisch.

## Fazit

- Eine Vielzahl an benignen Stimmlippenläsionen kann ambulant effizient und sicher therapiert werden.
- Photoangiolytische Laserverfahren bieten sich idealerweise vor allem bei vaskularisierten Stimmlippenalterationen an.
- Laryngeale Injektionen können sicher transnasal, aber ebenso transoral durchgeführt werden
- Laryngeale ambulante Eingriffe sollten nur dann als phonochirurgisch bezeichnet werden, wenn sie stimmdiagnostische Verfahren, aber auch die Option von stimmtherapeutischen Maßnahmen in das phonochirurgische Behandlungskonzept integrieren.
- Die ambulante Phonochirurgie *ergänzt* das operative Spektrum laryngealer Operationen in Vollnarkose.
- Notwendig sind ein systematisches Training transnasal endoskopischer Verfahren und die Beherrschung alternativer Techniken (transoral).
